# Arrearages—How to “Feel Better”

**Published:** 1880-12-20

**Authors:** 


### *ARREARAGES—HOW TO "FEEL BETTER."*

Notices will be found in the present issue, to those in arrears, requesting payment. Friends, the time is favorable for settling up. To those who have promptly and kindly responded to our appeal made in the last number, we return our thanks, and respectfully request all others to respond at once, that we may be able to close up the business of the year, and to meet the increased demands which culminate so heavily upon the Journalist at this season. Friends, don't put off the matter, but act at once, and when it is done you will feel better; the printers will feel better, the editors will feel better, the Journal will read better, your patients will get better, and you, as a Doctor, will be better.

We are pleased here, to give place to a somewhat amusing extract sent us by a medical friend, who was kind enough to call on another subscriber (his neighbor), and collect his subscription which he remitted with his own. Dr. Land, the party called on, made allusion to the dun in the last issue, and our facetious correspondent, who is also something of a poet, sends us in rhyme the substance of what he said, as follows:

"Said Doctor Land, that Journal Man
Has sent a dunning letter;
I will not fret but pay the debt,
And then I'll feel much better.

For well I know the book is low,
For twelve long months of pleasure;
'Tis filled with lots of useful dots—
Indeed it is a taeasure."

A friend in need it is in deed—
It always makes me wiser;
And none will fail the cash to mail,
But a Skinflint or a Miser

